# Upper Airway Obstruction in a Newborn: A Rare Cause of Respiratory Distress

**DOI:** 10.7759/cureus.22126

**Published:** 2022-02-11

**Authors:** Nuno Rodrigues Santos, Ricardo Mota, Américo Gonçalves, Jorge Spratley, Henrique Soares

**Affiliations:** 1 Neonatology and Pediatrics, Centro Hospitalar Universitário de São João, Porto, PRT; 2 Neonatology, Centro Hospitalar Universitário de São João, Porto, PRT; 3 Otorhinolaryngology, Centro Hospitalar Universitário de São João, Porto, PRT; 4 Otorhinolaryngology, Faculty of Medicine, University of Porto, Porto, PRT; 5 Obstetrics, Gynecology, and Pediatrics, Faculty of Medicine, University of Porto, Porto, PRT

**Keywords:** congenital, solitary median central incisor, respiratory distress, pyriform aperture stenosis, newborn

## Abstract

Newborns are considered obligate nasal breathers until the eighth week of life. Therefore, upper nasal obstruction in a newborn can present as a potentially life-threatening complication. Congenital nasal pyriform aperture stenosis (CNPAS) is a rare form of upper airway obstruction caused by a narrowing without occlusion in the most anterior opening of the bony nasal airways. According to the severity of this stenosis, early onset of respiratory symptoms can arise in the newborn.

In this article, we present the case of a male term newborn with no prior relevant family history and uneventful gestation delivered in a eutocic manner with an adequate transition to the extra-uterine environment. On his first day of life, progressive respiratory distress, inability to breastfeed, and impossibility to make nasogastric probe progress through both sides of the nose were observed, leading the newborn to be admitted to a neonatal intensive care unit.

During imagiological assessment with perinasal computerized tomography (CT) scan, an almost total occlusion of the pyriform aperture and a solitary median maxillary central incisor (SMMCI) were identified. Additional evaluation with brain magnetic nuclear resonance imaging (MRI) was unremarkable with no midline defects identified. Endocrine laboratory assessment was also normal.

The newborn underwent pyriform aperture permeabilization surgery via a sublabial approach with bilateral nasal stent introduction, enabling total resolution of the initial respiratory symptoms. No incurrences were reported during the post-operatory follow-up period.

With the present case report, the authors are trying to raise awareness for CNPAS not only as a rare cause of respiratory distress in the newborn but also as a clinical entity that can be associated with midline defects, which require further additional investigation and intervention.

## Introduction

Upper nasal obstruction in the newborn can present as a potentially lethal complication as a newborn is considered an obligate nasal breather until the eighth week of life [1,2]. Congenital nasal pyriform aperture stenosis (CNPAS) was first, clinically, described by Brown et al. in 1989. It is a rare form of upper airway obstruction with an estimated incidence of 1:25,000 cases [2]. It is caused by narrowing without occlusion in the most anterior opening of the bony nasal airways limited laterally by the nasal process of the maxilla, inferiorly by the horizontal process of the maxilla and the anterior nasal spine, and superiorly by the nasal bones [1-5].

Stenosis of this anatomic region can produce symptoms, immediately after birth, such as apnea, cyclic cyanosis, difficulty feeding, and respiratory distress [1-3]. In less severe cases, when a later diagnosis is made, it can also be responsible for the infant’s failure to thrive [1,6]. It is clinically suspected upon the impossibility to make a nasogastric probe progress through both sides of the nose [1-5]. The frequency of the symptoms and severity is usually proportional to the degree of the stenosis [1-3]. They can worsen in the presence of concomitant infection or during breastfeeding, and they can be relieved by crying [1].

CNPAS may not be initially diagnosed, and it can be mistaken with other more common causes of neonatal respiratory distress such as choanal atresia [6,7]. The diagnosis of pyriform aperture stenosis can be made with computerized tomography (CT) scan obtaining contiguous axial sections in a plane parallel to the anterior hard palate, showing a whole pyriform aperture width inferior to 11 mm [1,2,3,7]. However, normal whole pyriform aperture width is thought to range from 8.8 to 17.2 mm, and for this reason, some authors consider that measurements smaller than 8.0 mm should be termed as the inferior threshold to define abnormal [4-6].

CNPAS can be either present as an isolated morphogenic variant or can be associated with solitary median maxillary central incisor (SMMCI) that has been recognized as a microform of holoprosencephaly [8,9]. Thus, association with SMMCI requires further imagiological investigation with brain magnetic nuclear resonance imaging (MRI) to access the possibility of other underlying midline defects that together can reflect a developmental field defect [10-12].

The management of CNPAS depends upon the severity of the symptoms and the patient’s clinical evolution. While mild obstruction can be treated conservatively with intranasal corticoids or nasal decongestants for a few days, a moderate or severe obstruction will require surgical intervention [13].

This case report aims to highlight the importance of a timely diagnosis to prevent severe respiratory complications and the deleterious clinical evolution of the patient while raising awareness for other possible findings that should prompt additional investigation.

## Case presentation

We present the case of a male term newborn with irrelevant family history and uneventful gestation who was delivered in an eutocic manner in a private hospital with an adequate transition to the extra-uterine environment.

The first physical examination, immediately after birth, was unremarkable, and the neonate did not have any clear dysmorphic features. During initial breastfeeding, a clinical evident nasal obstruction without hypoxemia was first noted. Physical examination revealed the impossibility to make a nasogastric probe progress through both sides of the nose. The newborn was, immediately, transferred to the neonatal intensive care unit (NICU) of a central hospital with pediatrics and otorhinolaryngology support. He was breathing spontaneously upon admission, but during his first 24 hours of life, a sudden clinical deterioration with progressive respiratory distress and hypoxemia needing orotracheal intubation ventilatory support was soon noticed. Chest radiography and laboratory workup upon NICU admission were both normal.

**Figure 1:**
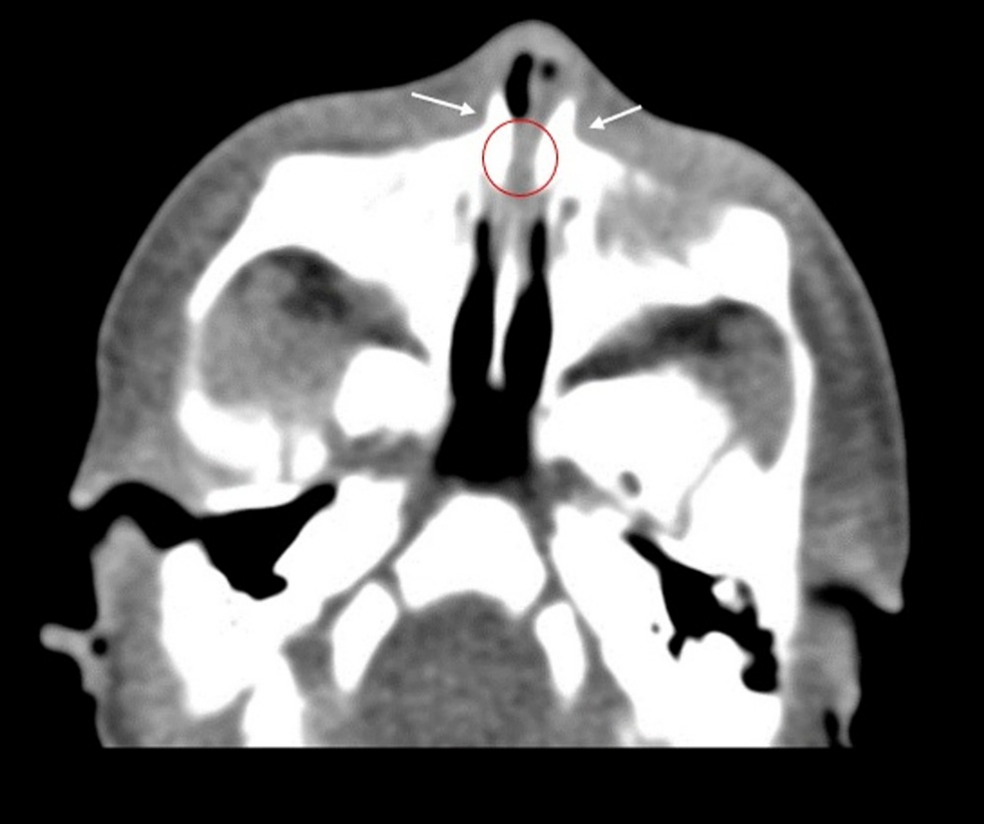
Axial CT scan shows a medial approximation of the nasal processes of the maxilla (arrows) causing a marked pyriform aperture narrowing and pyriform sinus hypoplasia (pointed out by white arrows and red ellipse)

**Figure 2:**
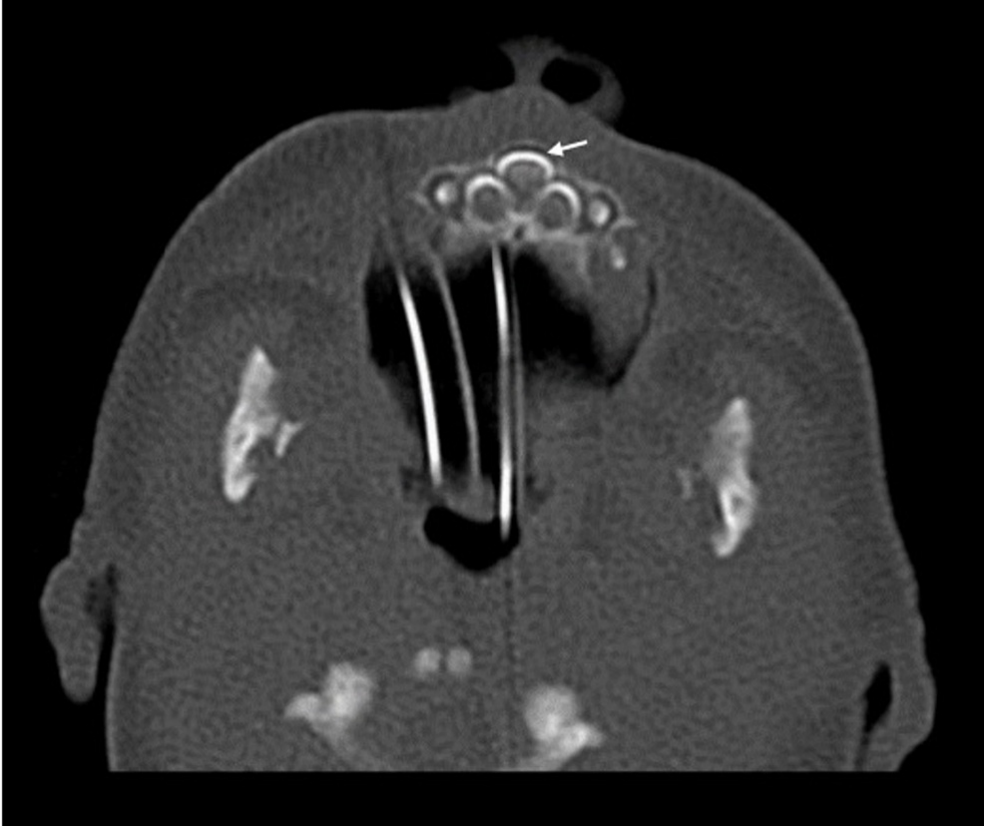
Solitary median incisor tooth (pointed out by white arrow) visible in axial CT scan

Further imaging study through a non-contrast CT scan of the paranasal sinuses confirmed the diagnosis of pyriform sinus hypoplasia (Figure 1), and an unerupted SMMCI was identified as well (Figure 2). Flexible nasal endoscopy was performed, and it showed an almost complete stenosis of the pyriform aperture with a small bilateral remaining slit that precluded further progression to the nasal fossae.

A later MRI performed at six days of life excluded any associated central nervous system malformations. Analytical study with endocrine assessment was performed at this time and deemed normal. Given the radiological findings and their correlation with the physical examination findings of severe stenosis of the pyriform aperture, the newborn’s family accepted to proceed with surgical repair.

**Figure 3:**
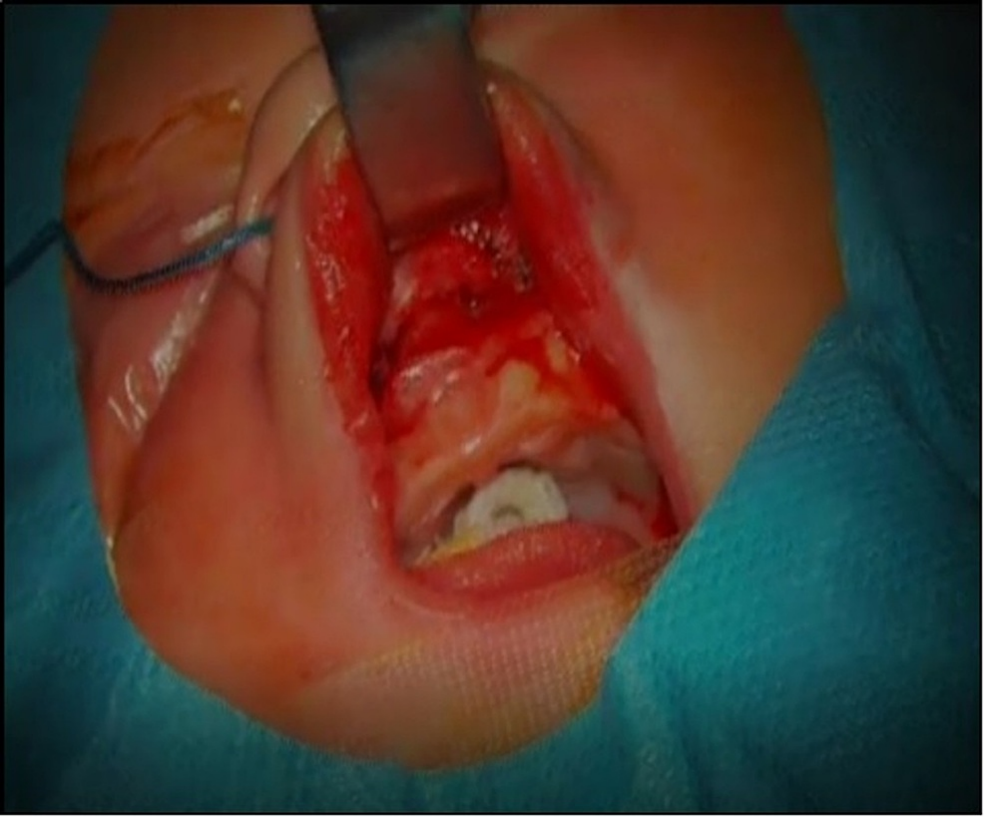
Pyriform aperture permeabilization surgery via sublabial approach with bone drilling and calibration

**Figure 4:**
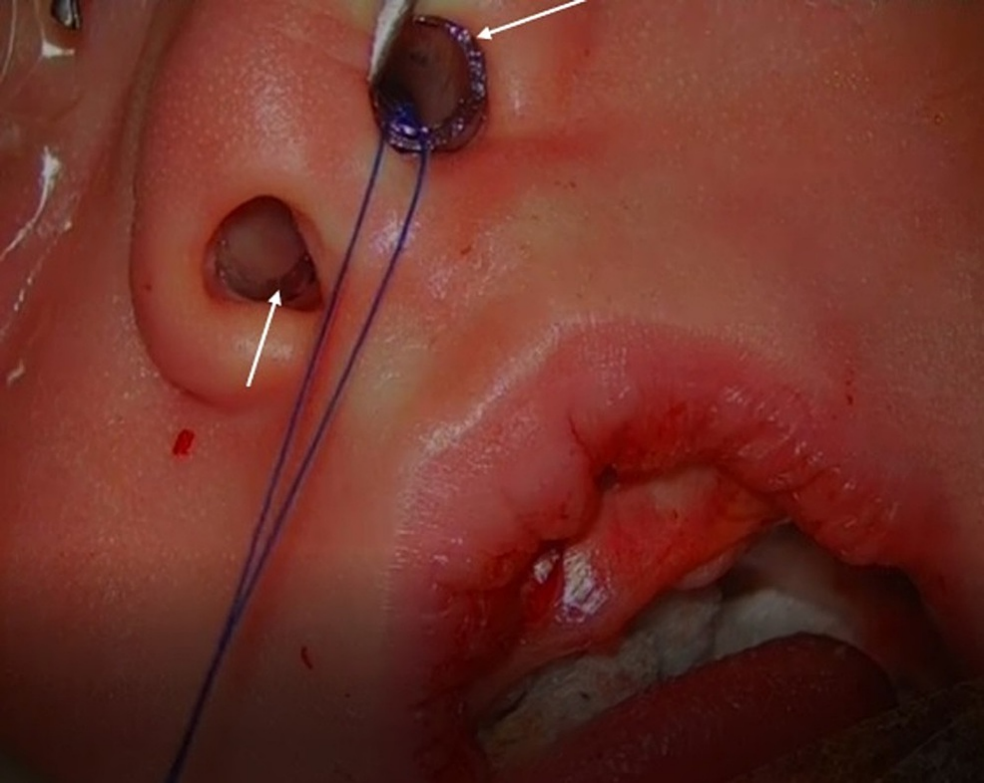
Insertion of nasal stents made from endotracheal tubes (pointed out by white arrows)

On the seventh day of life, the newborn was submitted to pyriform aperture permeabilization surgery via sublabial approach with bone drilling and calibration (Figure 3), followed by insertion of bilateral nasal stents made from endotracheal tubes (Figure 4). The surgery underwent uneventful. On the 22nd day of life, the nasal stents were removed, enabling full pyriform aperture’s permeability and total resolution of the symptoms initially present at birth.

**Figure 5:**
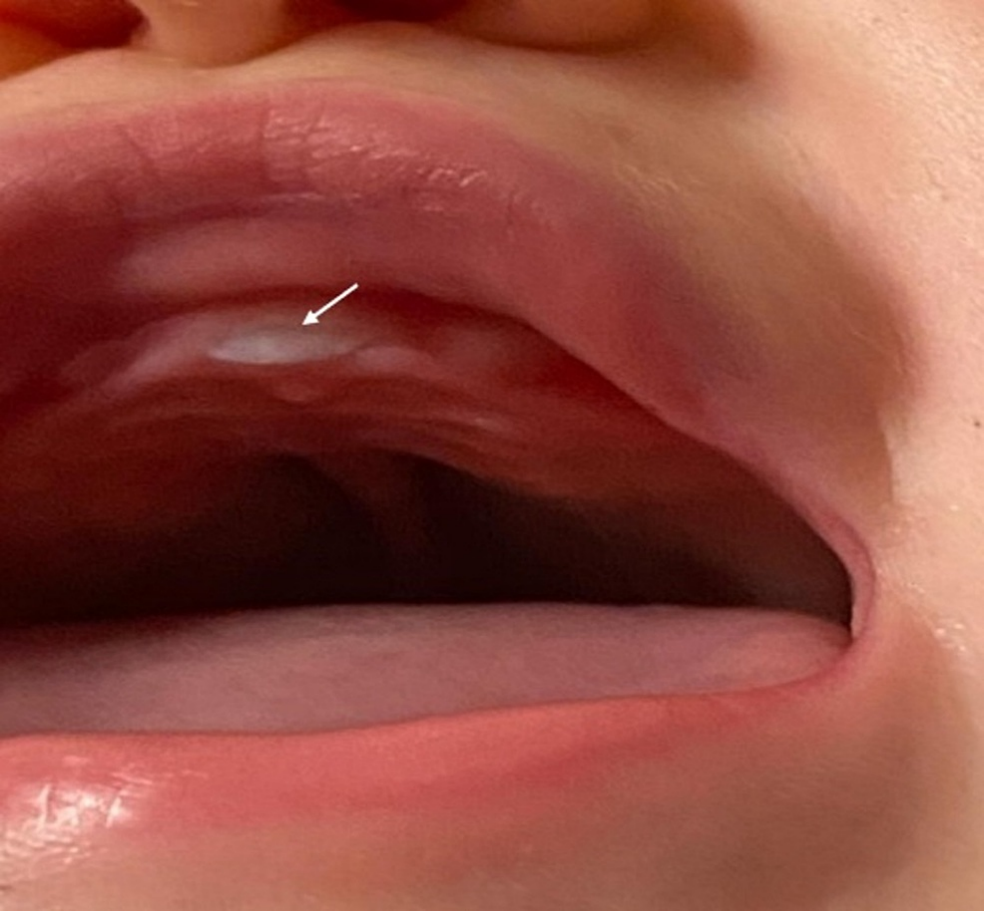
Erupted solitary median maxillary central incisor tooth (pointed out by white arrow)

The patient is currently eight months old and under ambulatory follow-up with regular medical consults in pediatrics and otorhinolaryngology. Figure 5 shows the solitary central incisor that has already erupted in the meantime. Until now, the infant registers a completely normal development without any relevant clinical incurrences to be reported.

## Discussion

Upper airway obstruction in the newborn can lead to severe respiratory distress because newborns are considered obligate nasal breathers [1,2]. Due to this fact, independent of the underlying etiology causing the obstruction, a timely diagnosis and intervention are mandatory to prevent life-threatening complications [1-5].

CNPAS is a rare clinical entity that must be considered in the differential diagnosis of pathologies that cause bilateral nasal obstruction, such as atresia or choanal narrowing, nasal trauma (hematoma and/or septal deviation), skull base malformations (meningoencephalocele and encephalocele), nasal tumors (rhabdomyosarcoma, hemangioma, glioma, and dermoid cyst), and nasal hypoplasia [1,6,7].

The pyriform aperture (bony inlet) is the narrowest part of the normal nasal airway; thus, any small changes in its cross-sectional area can result in a significant increase in the nasal airway’s resistance [5]. Due to this increased resistance, air passage can be compromised, and the newborn will present symptoms such as apnea, cyanosis, respiratory distress with or without hypoxemia, and difficulty while breastfeeding [3-5].

Two theories are currently adopted related to the pathogenesis of CNPAS [4,5,7]. The first one is based upon a deficiency of the primary palate, associated with a triangular hard palate [4,5]. The second one is based on the bony overgrowth in the nasal process of the maxilla, with a normal-shaped palate [4,5,7]. To access these morphological and structural variants involved in the physiopathology of CNPAS, a CT scan is required. A whole pyriform aperture’s width with less than 11 mm obtained by CT scan with thin contiguous axial sections in a plane parallel to the anterior hard palate in a term infant is considered confirmative of CNPAS diagnosis [1,2,3,7].

In our clinical case, the newborn was born without any immediate respiratory symptoms, and no craniofacial dysmorphism was observed upon physical examination. The CT scan was then performed because of the clinical deterioration that occurred in the first 24 hours of life. It showed severe pyriform sinus hypoplasia that was, furthermore, confirmed with flexible endoscopy showing almost full occlusion of the pyriform aperture. CT scan was not suggestive of a triangular hard palate. Henceforth, the bony overgrowth in the nasal process of the maxilla was assumed as the major etiological contributor to our patient’s CNPAS.

During the diagnostic workup of CNPAS, CT imaging also revealed an SMMCI tooth. This finding is a part of a syndrome called SMMCI, which represents a spectrum of midline facial defects and other possible congenital anomalies [9-11]. It has an estimated incidence of 1:50,000 live births, and its phenotypic characteristics at birth are an arch-shaped appearance of the upper lip, prominent maxillary alveolus, absent labial frenulum, narrow nose, and “V”-shaped palate with a prominent narrow ridge along the midpalatal suture, all of which were not present in our patient [11].

According to the international literature, the association of CNPAS with an SMMCI occurs in 40%-75% of CNPAS cases [8,9]. The presence of CNPAS with SMMCI raises awareness for other concomitant midline intracranial defects such as holoprosencephaly, microcephaly, hypoplasia of the corpus callosum, midface hypoplasia, pituitary dysfunction, submucous cleft palate, and chromosomal abnormalities [9,12]. Additional investigation with brain MRI was performed in our patient, and no midline defects were detected.

Endocrine abnormalities can be present in up to 40% of the cases with hypothalamic-pituitary axis (HPA) defects such as ectopic pituitary glands, hypothalamic agenesis, pituitary hypoplasia, or pituitary aplasia [9,11,12]. Consequently, patients with CNPAS and identified HPA defects can show abnormal production of growth hormones (GHs), adrenocorticotropin hormone (ACTH), anti-diuretic hormone (ADH), cortisol, and insulin-like growth factor (ILGF), which may require early endocrinologic repletion therapy [9,12].

The lack of the previously mentioned hormones and factors can lead to short stature, neonatal hypoglycemia, compromised stress response, and abnormal growth and development [12]. Laboratory endocrine study was performed on our patient for all the previously mentioned hormones and factors, and all the results were unremarkable. Hypoglycemia was never present during hospital in-stay.

Chromosomal abnormalities such as rings 18p-, 13p-, 3p+, 5q, and 7q- have also been described in international literature as being associated with CNPAS, either in its isolated form or associated with other clinical findings [9-11]. In our clinical case, genetic testing was also performed, and no chromosomal abnormalities were identified.

Once the diagnosis of CNPAS is established, conservative treatment involving the use of topical nasal decongestants, humidification, and lavage feeding is the initial line of management [13,14]. Surgical treatment aimed at the widening of the bony inlet is performed only when conservative management fails [13,14]. According to Rao et al., diameters inferior to 5 mm of the width of the whole pyriform aperture (confirmative of a moderate to severe stenosis) and patients unresponsive to conservative treatments are the main criteria that define surgical treatment [13]. The surgical procedure involves pyriform aperture enlargement through an endo-oral sublabial approach to reshape the stenotic area with burs [13]. After surgery, the use of nasal stents with posterior removal reduces the recurrence of CNPAS and scar-related stenosis [13].

In our patient, who registered a fast clinical deterioration with the need for orotracheal intubation for mechanical ventilation, conservative management was not an option. After confirming the diagnosis, surgical intervention was necessary to correct the severe pyriform aperture stenosis, and it went without incurrences.

In an international study by Pérez et al., where a series of 19 patients (ranging from 15 days of life and seven-year-old patients) underwent CNPAS surgery, 47.6% known syndrome association was reported [14]. The authors referred to one patient with congenital toxoplasmosis, two with associated VACTERL (vertebral defects, imperforate anus, cardiac defects, tracheoesophageal fistula, renal anomalies, and anomalies in limbs), one with Duane syndrome, one with midline syndrome, three with chromosomopathies, and one with plagiocephaly at the time of surgery [14].

According to Gonik et al.'s systematic review, the overall success of surgery with a single intervention was 85.7% [15]. In the presence of patients with identified syndromic associations, the rate of surgical failure was 36.8% [14,15].

## Conclusions

CNPAS is a rare cause of nasal airway obstruction, which can mimic choanal atresia in a neonate. Early identification of CNPAS is crucial to define proper management (either conservative or surgical). Recognition of the normal morphology of a neonate’s nasal cavity is essential to achieve a correct and prompt diagnosis. A whole pyriform aperture of width less than 11 mm in a CT scan confirms CNPAS in a full-term neonate.

In the presence of an SMMCI, considered as a microform of holoprosencephaly, physicians should proceed with additional imagiological studies to access the additional midline defects. Endocrine laboratory assessment should be performed in patients with CNPAS associated with SMMCI to exclude hypopituitary deficiency with analytical repercussions that may require hormonal therapy. Conservative or surgical management will depend upon the dimension of the pyriform’s aperture described in the CT scan and the patient’s respiratory symptoms and clinical evolution.
